# Determinants of Investment Behavior in Mutual Funds: Evidence From Pakistan

**DOI:** 10.3389/fpsyg.2021.666007

**Published:** 2021-07-12

**Authors:** Sharaz Saleem, Faiq Mahmood, Muhammad Usman, Mohsin Bashir, Rizwan Shabbir

**Affiliations:** ^1^Lyallpur Business School, Government College University, Faisalabad, Pakistan; ^2^Department of Management Sciences, University of Gujrat, Gujrat, Pakistan

**Keywords:** mutual fund investors behavior, investment criteria, mutual fund awareness, risk perception, return perception, financial literacy

## Abstract

This paper aimed to provide empirical evidence on the behavior of the investor toward mutual funds by considering its relationship with risk perception (RP), return perception (Return P), investment criteria (IC), mutual fund awareness (MFA), and financial literacy (FL). Data were collected using a questionnaire from 500 mutual fund investors, from which 460 questionnaires were used for the analysis. In addition, the snowball sampling technique was used to collect data from different cities in Pakistan. The result showed that RP, Return P, and MFA are insignificant and negatively affect the behavior of mutual fund investors. Investment criteria have a negative and significant effect on the behavior of mutual fund investors. Financial literacy has a positive and insignificant effect on the behavior of mutual fund investors. The results provide better information and guidance to investors and policymakers on the factors that affect the behavior of mutual fund investors.

## Introduction

A mutual fund is one of the professionally well-managed portfolios that typically pool funds for purchasing different shares from a variety of investors. Mutual funds utilize the wealth of small investors and families and make them available in the form of shares in business avenues, such as securities, bonds, and other financial instruments, to the economy. Mutual funds are very important for any market operation since risk control is also the most important issue, and it is important to analyze multiple variables that impact investors. In addition, the mutual fund makes it easier for small investors who do not have adequate knowledge, expertise, and low-risk tolerance to invest their savings in profitable portfolios by more skilled fund managers. To achieve a return for clients, these skillful technical managers target profitable and outperforming financial instruments.

In 1774, when the nation faced a massive downturn in its banking industry, the first mutual fund was launched in the Netherlands, followed by North America in 1924, and since 1980, the mutual fund has been a critical investment pool around the world. The mutual fund was first established in Pakistan in 1962 by the investment corporation. The Pakistan mutual funds association is described to be using mutual funds as a pooled investment system using a holistic approach (Ahmed and Siddiqui, 2020). Mutual fund investing is a risky investment operation. This could be due to poor production of the asset class, change in design, and increases in high return costs. Sometimes the loss can also be related to the shift of the successful fund manager, which will allow you to generate a profit (Kaveri and Bindu, 2017). Earlier research indicates that people with low-level financial literacy (FL) face personal financing issues such as savings, lending, investments, and pension plans. Hence, the judgment on investment means that particular transactions include risks and revenues, and a certain intensity of FL is needed to understand the risks and revenues involved with such purchases (Assefa and Durga Rao, 2018).

The investment behavior of an individual may be studied under the theory of planned behavior as an extension of the theory of reasoned action. The theory of reasoned action if that purpose is the immediate falling of their behavior. It suggests that the behavior of an individual, which in turn is the responsibility of the attitude to an active and subjective norm, should be governed by his/her behavioral intention (Kaur and Kaushik, 2016). This investing scheme is meant for those who want to invest in securities such as equity, stocks, instruments of the money market, and related assets. Mutual funds, especially in recent years, are an increasingly favored investment mode; this is apparent from the growing number of AMC funds. In 2018, the overall asset management companies (AMCs) stood at 20 with 248 funds under management with an average percentage shift of 40% for the year. The number of newly launched funds is another development that reflects the boom in the mutual fund market, with open funds facing rapid growth. According to the latest data released by the mutual fund association of Pakistan, the current amount of funds under administration as of December 2018 is Rs. 584 billion compared with Rs. 408 billion in 2014, reflecting a significant rise of 43% in 5 years. The tremendous number of mutual fund investors worldwide, especially in developed countries, indicates the option of investment. With the value of mutual funds and growth opportunities outlined above, mutual funds are becoming the center of focus, especially for researchers. Different authors conducted various facets of mutual funds, such as the effect of marketing with passively and actively managed funds, participation in foreign funds, and many more (Ahmed and Siddiqui, 2020).

According to Kaur and Kaushik (2016), perception has two dimensions: risk perception (RP) and return perception (Return P). First, perception is a method through which people look, evaluate, identify, and respond to all types of environmental information. Second, perception is a process through which a person tries to explain sensory data preeminently to help facilitate the participant to make a final verdict based on his intensity of experience and experience. The definition of “risks perception” applies to how investors perceive the risk of financial assets according to concern and experience. Risk perception implies an assumption that a risk arising is unbiased or incoherent or the degree, extent, and timing of its consequences retained by a person, group, or society is a significant success factor in facilitating good decision-making in risky situations. The fact that every investor has a tolerance to risk and RP complicates the study of financial risk. Therefore, a major factor that affects investment decisions is the RP of investors (Sindhu and Kumar, 2014).

Investment criteria (IC) consist of defined parameters for evaluating the acquisition goal valued by financial and strategy purchasers. Sophisticated buyers typically have two criteria sets: the requirements that are revealed to brokers and investment bankers publicly so that they know how the user is looking to make appropriate contracts and the parameters for internal review that have been established to allow buyers to make quick decisions on whether the transaction should be pursued more closely. Geographical, investment size, or targeting business and industry are the most common publicly disclosed investment requirements. Several investors often disclose requirements regarding the type of investment, including management buyouts, distressed assets, and circumstances of succession. Investment criteria are generally defined as “another equity, which means that investments with the highest turnover rate on capital investment should be selected” (Chenery, 1953).

Awareness programs, especially for institutional investors, are quite necessary to reduce the effect of interrogative or gambler mistakes (Abbas et al., 2019). The research clarified the awareness of investors regarding mutual funds, the perceptions of investors, their priorities, and the level of satisfaction with mutual funds. This study aimed to know about this intensity of awareness for mutual funds and about investor preferences for mutual funds (Sehdev and Ranjan, 2014). Financial literacy can also be described as integrating the knowledge of investors about, and their responsiveness to, financial risks, financial opportunities, informed decisions, information on where to support, and other successful steps to enhance the financial well-being of financial instruments and principles (Abdeldayem, 2016).

The study aimed to (i) determine the choice of the investor on multiple forms of investment in Pakistan; (ii) find the effect of RP on investment behavior of investors toward the mutual fund in Pakistan; (iii) recognize the effects of the perception of return on the investment behavior of investors in Pakistan against mutual funds; (iv) identify the effects of mutual fund awareness (MFA) on the investment behavior of investors in Pakistan; (v) identify the influence of investment parameters on the investment behavior of investors in the mutual fund of Pakistan; and (vi) identify the effects of FL on the investment behavior of investors in the mutual fund of Pakistan.

The significance of this investigation is manifold. The research will cover several key factors of the investment behavior of mutual funds within the Pakistan mutual fund industry. First, it is imperative to appraise FL and its link with investment behavior; moreover, not much research was conducted in Pakistan to identify this relationship. The present review would provide a momentous contribution to behavioral finance through insights into the relationship between FL and investment behavior. This research aimed to identify the components that persuade the investment behavior of investors toward mutual funds. It also focused on the impact of awareness, FL, investment, and perception of investors on their investment behavior toward the mutual funds in Pakistan. As per our knowledge, no research has been conducted in Pakistan using the same variables used in this study.

This research includes new areas such as awareness of the behavior of mutual fund investors toward mutual funds, which has not been influenced in Pakistan, and no systematic study has been done on the behavior of investors in mutual funds. Therefore, this article would add to quality literature on behavioral finance, in particular on the behavior of investors against the mutual fund. The rest of the study is organized as follows. Section Literature Review presents the literature review. Section Methodology outlines the methodology of the study, detailed data source measures, and methodology deployed to test the various hypotheses. Section Results and Discussion provides empirical findings of the study. Finally, Section Conclusion presents the concluding remarks.

## Literature Review

According to the base of this study, the research on the determinants of the behavior of the investor in mutual funds shows that evidence from Pakistan could be categorized into two parts: mutual funds and investors. These studies focus on mutual funds to examine the effect of different variables on the behavior of the investor in mutual funds. Also, they generally focus on variables like IC, RP, Return P, MFA, and FL. Examples of some studies related to those variables are given below.

The investment behavior of an individual may be studied under the theory of planned behavior as an extension of the theory of reasoned action. The purpose is to establish an immediate backdrop for the behavior. The theory suggests that the behavior of an individual, which is the attitude to an active and subjective norm, should be governed by his/her behavioral intention (Kaur and Kaushik, 2016). The theory of planned behavior explains the relationship between perceived behavioral control and intentions as this study setting is based on perceived behavioral control and its positive association with intentions (MFA, IC, RP, Return P, and FL).

Prospect theory is a behavioral theory that explains how individuals choose between risky and uncertain options (e.g., percent likelihood of gains or losses). It shows that people consider predicted utility about a reference point (for example, current wealth) rather than absolute outcomes. Thus, according to prospect theory, people are loss-averse, which was established by framing uncertain options. Since people fear losses rather than equal gains, they are more likely to take risks to prevent a loss. This hypothesis corresponds to the following trend with risk due to biased weighting of probabilities (see certainty/possibility effects) and failure aversion (Kahneman and Tversky, 1979; Kahneman et al., 2011).

The investing behavior of individual investors is very different from that of institutional investors. Individuals prefer to spend comparatively more in terms of non-tradable properties, such as real estate, hedge funds, or structured goods. The term institutional investor is commonly used to describe an entity, such as a mutual fund, a hedge fund, or a charitable organization, that invests on behalf of others. According to this report, investor behavior is one that an investor demonstrates in the quest for the acquisition, use, assessment, and disposal of products, resources, concepts, or experience to fulfill their needs and wishes. Environmental conditions largely impact the behavior of investors. While these variables are uncontrollable by the markets, they are quite significant in deciding the behavior of an investor. Investor actions, thus, suggest that investors modify their behaviors by purchasing and selling shares/commodities in different conditions (Elankumaran and Ananth, 2013).

Cognitive capacity are features that let a person perceive even before their occurrence to take consolidated action in advance. The cognitive and decision-making processes are significantly connected. This study has also shown that rational thought always seeks to be influenced by cognitive capabilities. The intensity of motivation and possible motivation enhance the cognitive capacity to face a challenging situation (Sarfraz et al., 2020a). This study looks at the behavior of the investor to identify better investment paths. Investment strategy is a program intended to assist an individual in choosing the best investment portfolio to benefit them in meeting financial targets within a specific period. Particular investment forms give the lender, the business, and the community more advantages. This research explores the behavior of investors when considering multiple investment options (Mane and Bhandari, 2014).

This study was performed to establish investor understanding of mutual funds, define the source of the information that affects the decision to buy, and define the factors that influence the choice of a particular fund. Among other factors, the study reveals that income schemes and open-ended strategies are more favored than growth schemes and close-ended schemes under the prevalent market conditions. Investors are pursuing security in order of priority for principal, liquidity, and capital growth. Magazines and newspapers are the first sources of information that investors can read about mutual funds/systems and is a major distinguishing factor in choosing mutual fund strategies on investor operation. The study also points out that investors see mutual funds as commodity goods and AMCs and that the consumer product distribution model should be adopted to catch the demand. Various papers and brief essays have been published in financial dailies, periodicals, and technical and academic journals since 1986, illustrating the fundamental definition of mutual funds and highlighting their relevance in the stock market environment. These papers and essays cover numerous elements such as mutual funds control, investor perceptions, investor security, and mutual growth pattern (Bansal, 2014).

In the view of China, the intensity of the replacement of chief executive officers (CEOs) among poor state-owned companies is considerable. The tolerance of the government is also a strong source of bad performance. State-owned companies in China are allegedly managed extensively (Sarfraz et al., 2020b). There is a lack of research explicitly conducted to understand the investment behavior of mutual fund investors in Pakistan. Studies conducted on investment behavior in traditional finance have progressed far beyond the viewpoints of Markowitz, whereby investors (supposed to be the logical benefit maximizers) consider anticipated returns and risks on investment opportunities as the only deciding factors in their decision on investment (Mishra and Kumar, 2016).

The effectiveness of a mutual fund relies on the level of awareness and confidence of investors—the pattern of investment changes with education, age, occupation, and gender. The ambition of this inquiry is to assess the intensity of awareness among investors. The research in Tezpur was conducted with a dataset of 99 individuals. The study initiates that investors have little awareness of mutual funds. The awareness of candidates with different educational backgrounds and ethnicity was also significantly different. A study has been done to determine the awareness of mutual funds of an investor to recognizing the sources of information affecting investor decisions and the elements affecting their choices. The study shows that income structures and open systems are needed in the prevailing market environment rather than development schemes and closed systems. Investors are pursuing the protection of principal, profitability, and appreciations in order of importance. The key information sources in the procurement of mutual funds are newspapers and journals. Investment schemes being informed by, and in the service of, an investor, is the main factor (Chaudhary, 2016).

The study initiates that investment in mutual funds relates to investor behavior, which attracts investment in mutual funds. The opinions and perceptions of the investor were studied on several topics, including the variety of mutual fund schemes (Trivedi et al., 2017). According to a research study, Chinese state-owned enterprises have financing challenges, except for state-owned firms, that can be funded through a commercial group. In this perspective, it may be stated that state-owned firms are more vulnerable than non-state-owned firms (Sarfraz et al., 2020c).

The study reveals the perception of risk and returns on the mutual funds of the investor. The inquiry examines the perception of an investor on mutual fund risk, returns from mutual funds, transparency, and disclosed practices compared with other financial avenues. The study also demonstrated that mutual funds are not considered a high-risk investment. Igt examined the behavior of investors against mutual funds. The study shows that RPs, current asset distribution, venture losses, investment blend, fund aging capital base, original fund efficiency, investment mixes, and portfolio diversification of an investor have been the main contributors to switching funds in the fund families. The research examined the significant aspects of mutual funds that affected investor perception and investigated the perception gaps between large and small investors based on factors investigated. Investment, return, and future have shown significant factors in the perception of investors of mutual funds (Dhar et al., 2017).

This research is done on FL and its association with financial instruments and financial activity. It concludes that even though individuals are well aware of different financial instruments and have little impact on their financial behavior, they are of limited value in the case of FL. Various other researchers have indicated that psychological influences such as self-control, avoidance, and instant fulfillment are likely to be more associated with financial power than a lack of financial knowledge. Therefore, rather than teaching persons on the economic front, it is more important to understand these behavioral inclinations (Gupta et al., 2018). The perception of risk, the perception of return, criteria of investment are some of the variables discussed in current research. By computing these variables, FL and awareness are used in this research. Until now, very limited evidence is available for research by using these two variables. According to our knowledge, there is no research conducted in Pakistan using these variables. As a result, current research analyses the link between the perception of return, the perception of risk, criteria of investment, awareness, and FL of investor behavior toward investment in mutual funds in Pakistan. It contained questions for RP and Return P, criteria of investment, MFA, and FL. Information about social demographics were obtained through direct questions about age, education, gender, level of savings, marital status, professional education, and income.

Following are the hypotheses of the study.

**H1**: Risk perception has a negative effect on mutual fund investment behavior.

**H2**: Return perception has a negative effect on mutual fund investment behavior.

**H3**: Investment criteria have a negative effect on mutual fund investment behavior.

**H4**: Mutual fund awareness has a negative effect on mutual fund investment behavior.

**H5**: Financial literacy has a positive effect on mutual fund investment behavior.

## Methodology

The study is quantitative, and the type of data is primary. The data were collected through questionnaires containing questions on RP and Return P, criteria of investment, awareness, and FL. Information about social demographics were obtained through direct questions about age, education, gender, level of savings, marital status, professional education, and income. Questionnaires were distributed through emails, social networking sites (Facebook, LinkedIn, etc.), and personal meetings. Out of the 500 distributed questionnaires, 460 responses were considered complete and up to the criteria. The sample included from a selected portion of a population for analysis is 460, and it is also known as the population part. In the research, we run binary logistic regression because the dependent variable is categorical. The explanatory variable in experiments is the one that is exploited; the dependent variable is the one that is examined. We also used Cronbach's alpha to check the reliability of the variables. In this study, the non-probability sampling technique was used to select a sample, that is, snowball sampling. The snowball sample was used to evaluate or analyze recruits from respondents, and it is used when potential participants are difficult to classify. It is called “snowball sampling” since (in theory) more “snow” is collected on the road before the ball is rolled up and is bigger. For example, basic random sampling, where the chances are the same for every respondent being chosen, was not effective. Instead, to select participants, the researchers used their judgment. Measurement of all variables used in the study is mentioned in Table 1.

**Table 1:** Measurement of variables.

**Variables**	**Description**	**Citation**
Risk perception	Risk perception is an independent variable (IV). It measured the comparison of risk perception of investors about mutual funds along with other investment avenues.	Kaur and Kaushik, 2016
Return perception	Return perception is an IV. It also measured through the comparison of mutual funds investment perception along with other investment avenues concerning return perception.	Kaur and Kaushik, 2016
Investment criteria	Investment criteria is an IV. It is measured by the comparison of investment criteria factors along with the investors concerning mutual fund investments.	Kaur and Kaushik, 2016
Mutual fund awareness	Mutual fund awareness is an IV. It is measured by 13 statements of knowledge on a Likert scale.	Kaur and Kaushik, 2016
Financial literacy	Financial literacy is an IV. It is measured by financial attitude, financial knowledge, and financial behavior.	Gangwar and Singh, 2018

**Figure 1:**
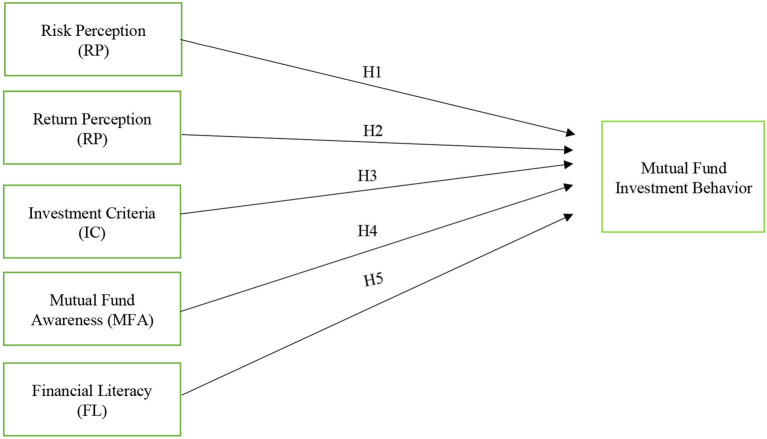
Theoretical framework. The interpretation of Figure 1 is given in the section Methodology.

The model formulated for regression analysis on basis of model Figure 1 is mentioned below

(1) $$\begin{array}{l} MFIB_{i,t}=\alpha_{0}+\beta_{1}{\left(IC\right)}_{i,t}+\beta_{2}{\left(MFA\right)}_{i,t}+\beta_{3}{\left(ReturnP\right)}_{i,t}\\ \;+\beta_{4}{\left(RiskP\right)}_{i,t}+\beta_{5}{\left(FL\right)}_{i,t}+\varepsilon_{i,t} \end{array}$$

MFIB, mutual fund investment behavior; IC, investment criteria; MFA, mutual fund awareness; Return P, return perception; RP, risk perception; FL, financial literacy.

Investment behavior is described as how the systems are judged, predicted, analyzed, and checked by investors. Decision-making involves the psychology of investing, collecting, identifying, and interpreting knowledge, study, and analysis. Variables used in the study are categorical. Criteria or guidelines under which the planning authority allocates the cumulative sum of the investible funds of the community to different channels are referred to as IC. Perceptions of risk and return are beliefs about the possibility of effect or loss. It is a subjective assessment of the features of risk and the severity of risk taken by individuals. Financial literacy refers to the knowledge and understanding of how money is earned, invested, and saved and the skills and abilities used to make choices by financial resources. These options include how much money to make, save, and spend.

## Results and Discussion

The demographic can be described in terms of analysis as a detailed group of people, organizations, institutions, and so on with shared themes of importance to the study. The shared features of the association distinguish them from others, organizations, objects, and so on. Individual investors have been included in the population from across Pakistan.

As shown Table 2, this research represents 83.26% of participants as male, 16.74% of them as female. This research represents 71.09% of the respondents as married and 28.91% as single. Individuals who fill the questionnaires have different education levels: 6.30% of respondents were matric pass, 43.26% were graduates, and 50.43% were postgraduates or higher during the survey. This research presents 48.04% of respondents having a professional education and 51.96% having no professional education. It also presents 11.96% of respondents as working in a government sector, 27.17% as running their own business, and 60.60% as working in the private sector. Also, 27.61% of the respondents save <10%, 39.35% save 11–20%, 19.78% save 21–30%, and 13.26% save above 30%.

Table 2Demographic and socio-economic characteristics of respondents.

**Table:** 

**A: DESCRIPTIVE**					
**Variables**	**Unit of measure (Individuals)**	**Frequency (*****N*****)**	**Percent**	**Valid percent**	**Cumulative percent**
Gender	Female	77	16.74	16.74	16.74
	Male	383	83.26	83.26	100.0
	Total	460	100.0	100.0	
Marital status	Married	327	71.09	71.09	71.09
	Single	133	28.91	28.91	100.0
	Total	460	100.0	100.0	
Education level	Matric	29	6.30	6.30	6.30
	Graduation	199	43.26	43.26	49.56
	Post-graduate, and higher	232	50.43	50.43	100.0
	Total	460	100.0	100.0	
Professional education	Yes	221	48.04	48.04	48.04
	No	239	51.96	51.96	100.0
	Total	460	100.0	100.0	
Occupation	Govt. employee	55	11.96	11.96	11.96
	Own-business	125	27.17	27.17	39.13
	Private-sector	280	60.60	60.60	100.0
	Total	460	100.0	100.0	
Monthly saving	<10	127	27.61	27.61	27.61
	11–20%	181	39.35	29.7	66.96
	21–30%	91	19.78	19.78	86.74
	Above 30%	61	13.26	13.26	100.0
	Total	460	100.0	100.0

**Table:** 

**B: QUANTITATIVE VARIABLES**				
**Variables**	**Minimum**	**Maximum**	**Mean**	**Std. deviation**
Age	18	52	30.38	6.617
Monthly income	10,000	400,000	56,982	50,619

In this research, those individuals who responded were between 18 and 52 years. Those individuals who responds to our survey their income level ranges from 10000 to 400,000.

Using Cronbach's alpha, the reliability of the measurements was assessed. Cronbach's alpha helps calculate the reliability of distinct groups and is the most famous test of internal consistency (“reliability”). It is most widely found in a survey/questionnaire with several Likert questions that shape a measure and decides if the scale is accurate. It includes tests of how much variation occurred in the ratings of multiple factors attributed to casual or random errors. As a general rule and a simple measure of building reliability, a coefficient greater or equal to 0.5 is deemed acceptable (Al-Tamimi, 2006). If you have inter-rater reliability issues.

The Cronbach's alpha (Table 3) of the five divisions, specifically, IC, RP, Return P, MFA, and FL, were 0.758, 0.765, 0.705, 0.705, and 0.893, respectively. The Cronbach's alpha indicates that all these divisions are appropriate.

**Table 3:** Reliability analysis.

**Variables**	**Cronbach's Alpha**
IC1	0.707
IC2	0.682
IC3	0.693
IC4	0.748
IC5	0.725
IC6	0.772
MFA1	0.758
MFA2	0.752
MFA3	0.747
MFA4	0.756
MFA5	0.734
MFA6	0.735
MFA7	0.749
MFA8	0.748
MFA9	0.749
MFA10	0.753
MFA11	0.758
MFA12	0.758
MFA13	0.758
RisP1	0.696
RisP2	0.681
RisP3	0.676
RisP4	0.668
RisP5	0.662
RisP6	0.686
RisP7	0.679
RisP8	0.682
RisP9	0.687
ReP1	0.696
ReP2	0.681
ReP3	0.676
ReP4	0.668
ReP5	0.662
ReP6	0.686
ReP7	0.679
ReP8	0.682
ReP9	0.687
FL1	0.889
FL2	0.888
FL3	0.890
FL4	0.888
FL5	0.889
FL6	0.888
FL7	0.890
FL8	0.887
FL9	0.890
FL10	0.888
FL11	0.887
FL12	0.891
FL13	0.891
FL14	0.888
FL15	0.888
FL16	0.889
FL17	0.890
FL18	0.889
FL19	0.890
FL20	0.891
FL21	0.889
FL22	0.889
FL23	0.891
FL24	0.890
FL25	0.889
FL26	0.889
FL27	0.891
Investment criteria (IC)	0.758
Mutual fund awareness (MFA)	0.765
Risk perception (RP)	0.705
Return perception (Return P)	0.705
Financial literacy (FL)	0.893
**Total**	**64**

In this research, we run the binary logistic regression because the dependent variable is categorical. The explanatory variable in the experiments is the one used; the dependent variable is the one that is examined. As shown in Table 4, IC insignificantly affect the behavior of the investor. According to Abbas et al. (2019), IC positively affect the behavior of the investors. On the other hand, IC have a negative and significant relationship with the behavior of the investor under the coefficient of −0.735, a result consistent with Kaur and Kaushik (2016), Abbas et al. (2019), Smith and Albaum (2013), and George and Mallery (2003). According to Kaur and Kaushik (2016), MFA has a positive effect on the behavior of investors. On the other hand, MFA has negative and insignificant relation with the behavior of the investor under a coefficient of −0.195, a result consistent with Chowdhury and Steve (2018). According to Abbas et al. (2019), RP and Return P negatively affect investor behavior. Risk perception and Return P both have a negative and insignificant association with investor behavior under the same coefficient of −0.010, a RP result consistent with Kaur and Kaushik (2016) and Abbas et al. (2019) and a Return P result consistent with Abbas et al. (2019) and Barlett et al. (2001). According to Gangwar and Singh (2018), FL has a positive and insignificant effect on the behavior of investors. Financial literacy has a positive and insignificant association with investor behavior with a coefficient of 0.287, a result consistent with results of Gangwar and Singh (2018). Per our knowledge, no research has been conducted in Pakistan using the same variables we used in this study.

**Table 4:** Variables in the equation.

		***B***	***S.E*.**	**Wald**	***df***	**Sig**.	**Exp(*B*)**	**95% C.I. for EXP(*****B*****)**	
								**Lower**	**Upper**
Step 1	Investment criteria	−0.753	0.212	12.625	1	0.000	0.471	0.311	0.713
	Mutual fund awareness	−0.195	0.497	0.154	1	0.695	0.823	0.310	2.182
	Risk perception	−0.010	0.231	0.002	1	0.967	0.990	0.629	1.559
	Return perception	−0.010	0.231	0.002	1	0.967	0.990	0.629	1.559
	Financial literacy	0.287	0.510	0.317	1	0.573	1.333	0.491	3.620
	Constant	1.963	1.083	3.283	1	0.070	7.122		

## Conclusion

This paper empirically investigated the determinants of the behavior of investors toward mutual funds in Pakistan. We established an important association between a mutual fund, the behavior of the investor, demographic characteristics of the respondents, and other variables used in the research. This research used a modified questionnaire that includes 64 items that fit into five divisions: IC, RP, Return P, MFA, and FL. Data were collected from 500 respondents, of which 460 questionnaires were used for the analysis. The logistic regression model was used to analyze the relationship between the variables. Cronbach's alpha was used to test the reliability of the data. Investment criteria is an important factor that affects the behavior of investors. If the investor has a high investment, then the association between IC and the behavior of the investor is positive and it should be negative when investment is low. In the study, the most important variable that affected the behavior of the investor is MFA. Awareness is one of the biggest reasons why investors invest in mutual funds. The behavior of investors depends on the awareness about mutual funds. If the investor has awareness about mutual funds, then there is a positive relationship between these two. If investors do have not much awareness of mutual funds, then the relationship is negative. The relationship between RP and investment behavior, whether positive or negative, depends on how investors perceive risk while investing. Return perception can affect the behavior of the investor in both negative and positive ways. It depends on how the investor perceives the return from investment. Financial literacy association with the behavior of the investor depends on the FL level of the investors. If the investor has a high level of FL, then the relationship is positive. If the investors have a low level of FL, then the association is negative. As per our knowledge, no research has been conducted in Pakistan using the same variables we used in this study.

## Implications

The research has implications for mutual funds and regulatory authorities. This inquiry identifies an inadequacy of awareness of mutual funds as a reason for the failure of mutual funds among certain sectors of society. Therefore, the popular funds and regulators need to concentrate their efforts on women, elderly groups, and middle-income groups to increase their knowledge of mutual funds.

The policy implications of this research are numerous. First of all, for fund managers of AMCs, our findings are compelling and insightful because they give further indication of the stimulus of Islamic and traditional funds performing in Pakistan. Furthermore, these results are also valuable for investors and provide them with important information about the characteristics of funds that undoubtedly improve performance.

## Limitations and Future Directions

It is suitable for investors who are aware of the professional competence of fund managers to join them to good return by moving to those funds. Investors should evaluate their portfolios on an ongoing basis and review their funds by modifying them as per position in the market to maximize returns.

The study was limited to 460 investors. The research has been conducted to analyze only some factors affecting the investment behavior of investors. The research is conducted only in a few cities. In this research sample, female existence is very low. This research enforced the technique of snowball sampling and may not be representative of the actual population. To educate investors, AMCs can organize seminars and training programs, among other activities, for investors, particularly in times of market fluctuations, economic recessions, market introduction of new products, etc. This will eliminate the uncertainty of the investor and create trust in the industry.

## Data Availability Statement

The raw data supporting the conclusions of this article will be made available by the authors, without undue reservation.

## Ethics Statement

Ethical review and approval was not required for the study on human participants in accordance with the local legislation and institutional requirements. Written informed consent for participation was not required for this study in accordance with the national legislation and the institutional requirements.

## Author Contributions

RS designed the model and the computational framework and analyzed the data. MU carried out the implementation. MB performed the calculations. SS wrote the manuscript with input from all authors. FM conceived the study and was in charge of overall direction and planning, supervised the findings of this work. All authors contributed to the article and approved the submitted version.

## Conflict of Interest

The authors declare that the research was conducted in the absence of any commercial or financial relationships that could be construed as a po tential conflict of interest.
